# Construction of two whole genome radiation hybrid panels for dromedary (*Camelus dromedarius*): 5000_RAD_ and 15000_RAD_

**DOI:** 10.1038/s41598-018-20223-5

**Published:** 2018-01-31

**Authors:** Polina L. Perelman, Rudolf Pichler, Anna Gaggl, Denis M. Larkin, Terje Raudsepp, Fahad Alshanbari, Heather M. Holl, Samantha A. Brooks, Pamela A. Burger, Kathiravan Periasamy

**Affiliations:** 1 Animal Production and Health Laboratory, Joint FAO/IAEA Division, International Atomic Energy Agency, Vienna, Austria; 2 Institute of Molecular and Cellular Biology and Novosibirsk State University, Novosibirsk, Russia; 3 Department of Comparative Biomedical Sciences, Royal Veterinary College, University of London, London, NW1 0TU United Kingdom; 4 Texas A&M University, College Station, Texas USA; 5 University of Florida, Gainesville, Florida USA; 6 Research Institute of Wildlife Ecology, Vetmeduni, Vienna, Austria

## Abstract

The availability of genomic resources including linkage information for camelids has been very limited. Here, we describe the construction of a set of two radiation hybrid (RH) panels (5000_RAD_ and 15000_RAD_) for the dromedary *(Camelus dromedarius)* as a permanent genetic resource for camel genome researchers worldwide. For the 5000_RAD_ panel, a total of 245 female camel-hamster radiation hybrid clones were collected, of which 186 were screened with 44 custom designed marker loci distributed throughout camel genome. The overall mean retention frequency (RF) of the final set of 93 hybrids was 47.7%. For the 15000_RAD_ panel, 238 male dromedary-hamster radiation hybrid clones were collected, of which 93 were tested using 44 PCR markers. The final set of 90 clones had a mean RF of 39.9%. This 15000_RAD_ panel is an important high-resolution complement to the main 5000_RAD_ panel and an indispensable tool for resolving complex genomic regions. This valuable genetic resource of dromedary RH panels is expected to be instrumental for constructing a high resolution camel genome map. Construction of the set of RH panels is essential step toward chromosome level reference quality genome assembly that is critical for advancing camelid genomics and the development of custom genomic tools.

## Introduction

The dromedary (single humped camel), with an estimated global population of 26.49 million^1^, is one of the most popular domestic species in regions with harsh climatic conditions. These animals predominantly inhabit arid and semi-arid areas that are not suitable for most crop and livestock production, mainly due to challenges of unpredictable rainfall and frequent occurrences of drought. Camels are mostly reared for milk, meat, draught, and racing and contribute significantly to the subsistence of many pastoral communities in Africa and Asia. As an adaptation to climate change, pastoralists in Africa who historically depended on cattle are shifting to camels due to their tolerance of severe droughts and ability to contribute to household nutrition and economy during dry periods^2,3^. Camel milk is fast gaining popularity across markets in many countries, with a good potential to support and improve the resilience of traditional pastoral systems^4–6^. In spite of the opportunities for sustainable camel production, systematic breeding for genetic improvement is constrained by several factors, such as lack of animal identification, performance recording systems, and modern genetic and genomic tools. Genetic resources for camelids (dromedaries and Bactrian camels, alpacas, and llamas) have been limited and developed only recently in the past ten years, lagging behind other livestock species.

Current genomic resources available for camelids include a comparative chromosome map of the dromedary with humans, cattle and pigs^7^, a whole genome cytogenetic map of the alpaca^8–10^, and genome assemblies at the scaffold level^11–13^. All camelids possess highly similar karyotypes with a rather high diploid number of 2n = 74, which particularly complicates chromosome identification and mapping^7,8^. There is no fine-scale high resolution mapping resources and/or chromosome level assemblies available for camelid genomes. However, availability of mapping resources will open up the opportunities for whole genome scans to identify selection signatures, perform linkage analysis, genome-wide association studies, comparative evolutionary genomics and development of genomic tools for breeding and improvement of camels. It has also been shown that a combination of RH panels with different levels of resolution produces superior maps^14,15^. In this paper, we describe the construction and validation of radiation hybrid panels for the dromedary, a useful genomic resource for studies in camelids.

## Material and Methods

### Animal sampling and ethics statement

A biopsy of ear skin tissue was obtained from a female dromedary camel named ‘Waris’ living at the first Austrian Camel Riding School in Eitental, Austria. The 6 mm biopsy punch was collected commensal during a diagnostic treatment for skin parasites by a veterinary surgeon following standard practices and the owner agreed to the usage of the sample for this study. Therefore, no further license from the “Ethics and animal welfare committee” of the University of Veterinary Medicine, Vienna, Austria was required. The pedigree of Waris showed that she was born to a dam of Canary Islands origin and a sire of North African origin. The whole genome sequence of Waris was previously published^13^ and the genome assembly is available in NCBI-GenBank (Bioproject: PRJNA269274; Assembly: GCA_008031251; Nucleotide: JWIN01000001-JWIN01035752). A second skin biopsy was collected from a male dromedary named CJ owned by Franklin Safari, Texas, USA. The 6 mm biopsy punch was collected following United States Government Principles for the Utilization and Care of Vertebrate Animals Used in Testing, Research and Training. The Informed Owner Consent Form for the procurement of blood and tissue samples from client-owned animals was approved by Texas A&M University Clinical Research Review Committee (CRRC 09–47). The 53X Illumina and 20X PacBio hybrid assembly of the CJ’s genome is being constructed^16^.

### Establishment of donor and recipient cells

The primary fibroblast cell line (CDR3) derived from ear perichondrium of female dromedary was established using standard methods of enzymatic tissue digestion^17^. The primary fibroblast cell line (CDR2) from male dromedary was established from the skin biopsy following the protocol of preparation of primary cultures using collagenase digestion^18^. The primary camel fibroblasts were propagated in AlphaMEM media with nucleosides and GlutaMAX supplemented with 15% FBS, 1X penicillin/streptomycin (100 units/mL, 100 µg/mL), amphotericin B (2.5 µg/ml), gentamicin (50 µg/ml), 1.5% AmnioMAX C-100 supplement and bFGF. Fibroblast cells from the third passage (CDR3) and from the sixth passage (CDR2) were used as donors while the thymidine kinase (TK^−^) deficient Chinese hamster cell line A23^19^ was used as the recipient. The recipient cell line was propagated in AlphaMEM media with nucleosides and GlutaMAX supplemented with 10% FBS, penicillin/streptomycin (100 units/mL, 100 µg/mL), amphotericin B (2.5 µg/ml) and gentamicin (50 µg/ml). The A23 cell line was also treated with 5-Bromo-2′-Deoxyuridine (BrdU) (0.03 mg/ml) one passage prior to fusion to remove TK^−^ revertants.

### Generation of 5000_RAD_ camel-hamster radiation hybrids

The generation of camel-hamster radiation hybrids was performed following the standard irradiation and cell fusion protocol adapted from Page and Murphy^20^. For the fusion experiment, 2 × 10^7^ female camel fibroblast cells (CDR3) were irradiated at 5000_RAD_ by gamma rays using a Cobalt-60 (^60^Co) source (Gamma Cell 220). Irradiated camel fibroblast cells were mixed with A23 hamster cells at two different ratios of 1:2 and 1:4 and fused using the incubation with polyethylene glycol (PEG, Cat. P7181, Sigma). Post-fusion, the cells were seeded onto 100 mm^2^ petri dishes at three densities to produce well-isolated colonies: 5 × 10^3^, 1 × 10^4^, and 5 × 10^4^ cells per dish (66 dishes total). The fused cells were grown in AlphaMEM media with nucleosides and GlutaMAX supplemented with 1 µM Ouabain, HAT, (100 μM hypoxanthine, 0.4 μM aminopterin, 16 μM thymidine), 10% FBS, penicillin/streptomycin (100 units/mL, 100 µg/mL), amphotericin B (2.5 ug/ml) and gentamicin (50 ug/ml). Ouabain (rodent cell line is less sensitive to toxic effect of ouabain) was used only during the first week post-fusion to eliminate intact camel cells that could have survived lethal irradiation. Hybrid colonies appeared after one week and were collected and transferred to 24-well plate using high vacuum grease (Cat. No. 15471977; Dow Corning Corp., MI, USA) and Pyrex^®^ 8 × 8 mm cloning cylinders (Cat. No. 3166-8, Corning Inc, NY, USA). After reaching confluency, the cells were transferred to 25 cm^2^ (T25) flasks and then passaged to two 150 cm^2^ (T150) flasks. After achieving confluency in T150 flasks, 4 million viable cells were cryopreserved in two vials, with 0.75 million used for preliminary DNA extraction and screening, while the remaining large pellet was kept for a final extraction of bulk DNA. The DNA from 0.75 million cell pellets was extracted in 96-well plate format using NucleoSpin^®^ 96 Tissue Core Kit (Macherey-Nagel, Düren, Germany) in an automated epMotion^®^ 5075 (Eppendorf, Hamburg, Germany). The bulk DNA was extracted from large cell pellets of selected clones using MasterPure™ DNA Purification Kit (Epicentre-Illumina, CA, USA). The final bulk radiation hybrid DNA was dissolved in Tris-EDTA buffer and stored at −80 °C.

### PCR based screening and scoring of radiation hybrids

A total of 48 primer pairs were designed using Primer 3^21^ to verify retention of donor genome in the camel-hamster radiation hybrids. To design the primer pairs, the target regions in camel genome were selected based on alpaca RH markers (Perelman *et al*.; unpublished). Selected marker sequences were searched against the whole genome shotgun sequence of Waris (NCBI Bioproject: PRJNA269274; NCBI Assembly: GCA_008031251 (Revised-CdRom64 assembly)) using NCBI MEGABLAST^22^. To ensure the primers were non-specific to hamster DNA, the target camel sequences were searched (blastn) against nucleotide collection (nr/nt) of the order Rodentia (taxid: 9989). In the few cases of hit sequences to *Cricetulus griseus*, we selected only markers that showed no matches to hamster sequence with camel primers based on Primer3. PCR was performed with 20 ng of DNA and 1.5 mM MgCl_2_ in 20 µl reactions under following conditions: initial denaturation for 5 min at 95 °C, followed by 35 cycles of denaturation for 30 s at 95 °C, 1 minute at specific annealing temperatures of marker locus, elongation at 72 °C for 1 min, and a final extension at 72 °C for 10 min. The details of primers along with expected amplicon size and annealing temperatures are presented in Supplementary Table ST1. Four out of 48 markers were excluded due to ambiguous PCR results. PCR screening of 186 radiation hybrids was performed twice for each of the 44 markers with the following controls: camel genomic DNA from the same donor cell line as a positive control, genomic DNA from hamster A23 cell line as a negative control, and water as a no template control. The PCR products were electrophoresed on a 2% agarose gel and scored manually as follows: 0 - No PCR product; 1 – Strong PCR product of the expected size; 2 – Weak PCR product of the expected size; 3 - Strong/weak PCR product of a similar but not expected size. Results from duplicate PCR screenings for each hybrid clone were combined to obtain a final score of positive, negative, or discordant as shown in Supplementary Table ST2. PCR repeatability was evaluated based on discordance (e.g. strong positive amplification of expected product during the first PCR, but no amplification during the second PCR) and difference (e.g. strong positive amplification during the first PCR, but weak positive amplification of expected product during the second PCR). The scoring was performed by a single evaluator for all markers.

### Generation and screening of high dose (15000_RAD_) camel-hamster radiation hybrids

Camel fibroblasts derived from the male dromedary CJ (CDR2) were irradiated at 15000_RAD_ using the same Cobalt source (Gamma Cell 220). For fusion, irradiated camel fibroblast cells were mixed with A23 hamster cells at two different ratios of 1:1 and 4:1. The fused cells were seeded onto 100 mm^2^ petri dishes with 1 × 10^4^, 5 × 10^4^ and 1 × 10^5^ cells per dish (81 dishes total) to produce well-isolated colonies. Subsequent culture and expansion of hybrid cells were performed similar to the 5000_RAD_ panel. PCR screening of 93 hybrids derived from 15000_RAD_ panel was also performed twice with the same set of 44 markers under the conditions mentioned in Supplementary Table ST1. Additionally, 15000_RAD_ hybrids were also screened for two other markers, TR4520 and TR5720, to ascertain the retention of dromedary Y-chromosome. The PCR products were electrophoresed on a 2% agarose gel and scored manually.

## Results

For the 5000_RAD_ panel, a total of 245 well isolated camel-hamster radiation hybrid clones were transferred into 24 well plates and cryopreserved, of which 186 were grown to confluent cultures in two 150 cm^2^ (T150) flasks. DNA extracted from the hybrid clones was used for PCR based screening of 44 marker loci distributed across 33 autosomes and the X chromosome. All of the 186 clones demonstrated strong amplification in at least one marker locus, suggesting retention of camel chromosomes. Among the markers tested, the mean RF per marker ranged from 14.5% (VOLP10) to 94.6% (JMJD6), with a mean of 49.6%. Higher retention frequency for markers located on chromosomal fragments containing dromedary camel scaffold 8666493 (e.g. JMJD6) was expected as it contains the marker gene (thymidine kinase) for post-fusion hybrid selection. Nevertheless, the mean RF per marker observed in the camel-hamster radiation hybrids was higher than reported for other species, including humans^23,24^. Further, certain marker loci showed higher discordance between duplicate runs, i.e. positive amplification in the first PCR and negative or non-specific amplification in the second PCR. Thirteen marker loci (KITL, GG_435, VOLP10, GNAQ, CMS13, CMS15, CMS9, GG_1032, GG_984, GATA4, GG_498, CREM, VOLP67) that showed discordant PCR results in more than seven out of 186 hybrid clones or high difference rate were excluded from further analysis. The remaining 31 markers were distributed across 25 autosomes and the X chromosome. The mean retention frequencies based on these 31 markers ranged from 3.2% to 93.5% per hybrid clone, with an overall mean of 50.3%. Among the 186 radiation hybrids, seven (3.8%) had a mean RF less than 10%, 17 (9.1%) had a mean RF ranging from 10–20%, 124 (66.7%) had a mean RF ranging from 20–70%, while 38 (20.4%) had a mean RF greater than 70%. Earlier reports based on simulation studies suggested that selection of hybrids having an overall mean RF of around 50% would be optimal, as hybrids retaining most or a few of the loci were equally uninformative for mapping, but radiation dose is an important factor to be considered too^25–28^.

**Figure 1:**
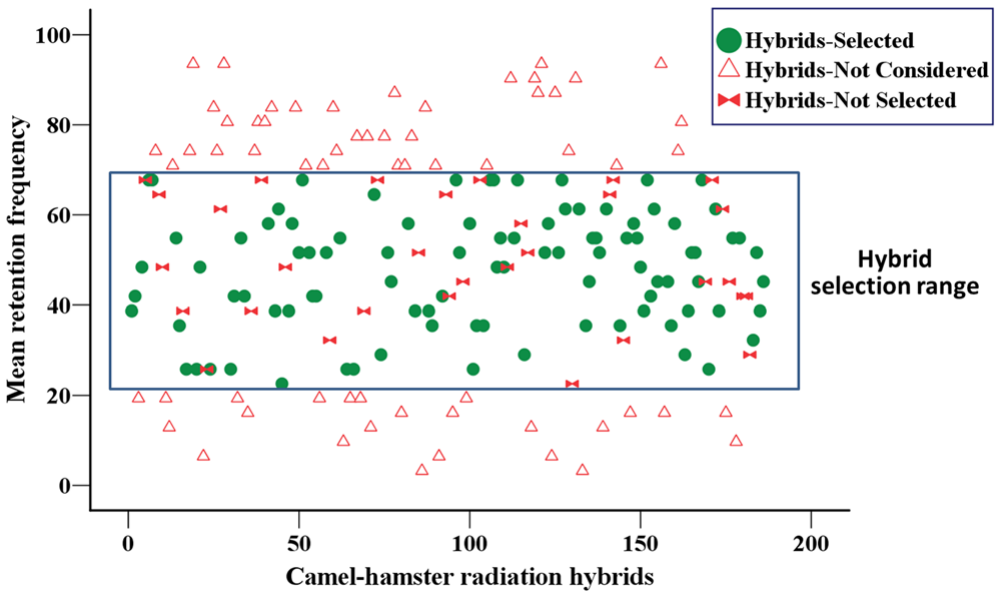
Frequency distribution of 186 camel radiation hybrids (5000_RAD_) based on the retention of donor genome.

In the present work, standard irradiation (5000_RAD_) was used to break the donor chromosomes and hence the collection of 124 hybrids that showed a mean RF range of 20–70% was considered for selection (Fig. 1). 93 hybrids in the above mentioned range that showed little or no discordance between two independent PCR screenings were selected. Additionally, those hybrids that showed more than six differences (not discordance; See Supplementary Table ST2) between duplicate runs were excluded. A high number of such differences suggest the presence of unstable or low copy fragments in the hybrids. Among the selected hybrids, 12 had a mean RF between 20–30%, 17 had a mean RF between 30–40%, 18 had 40–50%, 29 had 50–60% and 17 had 60–70%. The overall mean RF of the final camel 5000_RAD_ radiation hybrid panel was 47.7%, with a mean RF ranging from 22.6% to 67.7% per hybrid. The mean discordance and difference between repetitive screenings per selected hybrid was 0.007 and 0.082 respectively (Table 1).

**Table 1:** Retention frequency, stability of donor fragments, cell and DNA yield for the selected 5000_RAD_ camel-hamster radiation hybrids.

S.No.	Hybrid	RF	PCR repeatability		Hybrid yield		No. PCR
			Discordance	Difference	Cells (in million)	DNA (in µg)	
1	CDR3-10A	38.7	0.000	0.097	16.0	411.8	10295
2	CDR3-10B	41.9	0.000	0.097	13.0	293.1	7328
3	CDR3-11A	48.4	0.032	0.161	16.0	403.3	10082
4	CDR3-11C	67.7	0.032	0.161	8.0	269.8	6746
5	CDR3-12A	67.7	0.000	0.065	31.3	1055.4	26386
6	CDR3-13E	54.8	0.000	0.097	17.3	417.1	10428
7	CDR3-13F	35.5	0.000	0.129	33.0	979.5	24487
8	CDR3-14A	25.8	0.000	0.065	33.0	960.7	24018
9	CDR3-17B	25.8	0.000	0.065	22.0	558.6	13965
10	CDR3-18A	48.4	0.032	0.097	58.0	1321.1	33027
11	CDR3-19B	25.8	0.032	0.097	12.7	376.5	9413
12	CDR3-1D	25.8	0.000	0.000	21.3	629.3	15734
13	CDR3-1E	41.9	0.000	0.097	31.3	904.5	22613
14	CDR3-20A	54.8	0.032	0.032	17.3	424.0	10601
15	CDR3-20C	41.9	0.000	0.161	43.3	1096.0	27401
16	CDR3-21F	58.1	0.000	0.032	15.3	364.2	9104
17	CDR3-22C	38.7	0.000	0.097	7.1	482.8	12070
18	CDR3-22E	61.3	0.000	0.000	23.3	745.5	18637
19	CDR3-22F	22.6	0.000	0.032	23.3	784.7	19616
20	CDR3-23C	38.7	0.000	0.000	19.3	603.2	15080
21	CDR3-23D	58.1	0.032	0.032	25.3	426.5	10662
22	CDR3-24B	51.6	0.000	0.032	21.3	602.3	15057
23	CDR3-24C	67.7	0.000	0.032	6.1	205.8	5144
24	CDR3-25A	51.6	0.000	0.065	35.3	1621.6	40539
25	CDR3-25B	41.9	0.000	0.065	34.0	966.4	24159
26	CDR3-25C	41.9	0.000	0.032	37.0	994.4	24859
27	CDR3-2B	51.6	0.000	0.032	8.3	164.2	4105
28	CDR3-31B	54.8	0.032	0.065	10.0	180.6	4515
29	CDR3-32C	25.8	0.000	0.065	14.0	331.3	8282
30	CDR3-35A	25.8	0.000	0.000	12.0	373.0	9324
31	CDR3-39C	64.5	0.000	0.032	19.3	558.1	13951
32	CDR3-41A	29.0	0.000	0.032	6.3	360.5	9014
33	CDR3-41C	51.6	0.000	0.161	31.3	687.1	17178
34	CDR3-41D	45.2	0.000	0.065	35.3	807.1	20177
35	CDR3-43C	58.1	0.032	0.065	34.0	705.1	17627
36	CDR3-44B	38.7	0.000	0.032	33.0	724.5	18113
37	CDR3-45C	38.7	0.032	0.065	30.0	459.1	11476
38	CDR3-45D	35.5	0.000	0.065	33.0	1271.5	31787
39	CDR3-46A	41.9	0.000	0.000	17.0	398.6	9965
40	CDR3-47A	67.7	0.000	0.129	28.0	825.0	20626
41	CDR3-47B	51.6	0.000	0.097	16.0	778.2	19455
42	CDR3-48D	58.1	0.000	0.065	21.0	682.1	17053
43	CDR3-48H	25.8	0.032	0.097	40.0	1084.1	27103
44	CDR3-49A	35.5	0.000	0.065	62.0	841.9	21047
45	CDR3-4A	35.5	0.000	0.065	25.3	474.5	11862
46	CDR3-4C	67.7	0.000	0.000	15.3	395.3	9883
47	CDR3-4E	67.7	0.000	0.065	11.9	283.8	7096
48	CDR3-50A	48.4	0.000	0.161	30.0	632.9	15822
49	CDR3-50C	54.8	0.000	0.097	41.0	553.0	13826
50	CDR3-52A	48.4	0.000	0.129	24.0	788.8	19719
51	CDR3-54A	54.8	0.000	0.129	22.0	417.3	10433
52	CDR3-54B	67.7	0.000	0.032	7.0	304.4	7610
53	CDR3-54D	29.0	0.000	0.065	25.0	574.3	14356
54	CDR3-56D	51.6	0.000	0.161	31.3	824.5	20612
55	CDR3-57A	58.1	0.000	0.065	21.3	887.4	22186
56	CDR3-58B	51.6	0.000	0.065	15.3	512.3	12808
57	CDR3-58C	67.7	0.000	0.032	21.3	763.4	19085
58	CDR3-58D	61.3	0.000	0.097	10.5	393.2	9831
59	CDR3-5A	61.3	0.000	0.129	17.3	656.9	16422
60	CDR3-5C	35.5	0.000	0.097	27.3	876.3	21907
61	CDR3-60B	45.2	0.032	0.194	23.3	571.2	14280
62	CDR3-60C	54.8	0.000	0.161	15.3	472.3	11808
63	CDR3-60E	54.8	0.000	0.032	17.3	513.3	12832
64	CDR3-61A	51.6	0.000	0.032	13.0	319.3	7983
65	CDR3-61E	61.3	0.000	0.097	18.0	777.9	19448
66	CDR3-63C	35.5	0.000	0.161	14.0	316.7	7917
67	CDR3-63E	54.8	0.000	0.032	31.3	826.4	20659
68	CDR3-65C	58.1	0.000	0.129	12.0	319.9	7997
69	CDR3-65F	54.8	0.000	0.097	43.0	775.9	19398
70	CDR3-65G	48.4	0.000	0.129	37.0	1359.4	33985
71	CDR3-66B	38.7	0.000	0.097	35.0	967.2	24181
72	CDR3-66C	67.7	0.000	0.065	8.0	220.0	5501
73	CDR3-67D	41.9	0.000	0.129	29.3	619.6	15490
74	CDR3-68A	61.3	0.000	0.065	49.3	1013.7	25343
75	CDR3-68B	45.2	0.065	0.097	17.3	629.3	15733
76	CDR3-6B	45.2	0.000	0.129	57.3	890.0	22250
77	CDR3-6C	35.5	0.000	0.097	33.3	836.2	20906
78	CDR3-6D	58.1	0.000	0.129	15.3	331.0	8275
79	CDR3-71B	29.0	0.032	0.065	37.0	934.8	23369
80	CDR3-72A	38.7	0.000	0.097	36.0	1074.5	26863
81	CDR3-72B	51.6	0.032	0.129	21.0	606.4	15161
82	CDR3-72C	51.6	0.000	0.129	19.0	432.2	10804
83	CDR3-73A	45.2	0.000	0.097	15.0	358.8	8969
84	CDR3-73B	67.7	0.032	0.161	19.0	649.1	16228
85	CDR3-74A	25.8	0.032	0.097	33.0	859.5	21487
86	CDR3-74C	61.3	0.000	0.065	28.0	928.7	23218
87	CDR3-75A	38.7	0.000	0.065	14.0	442.9	11073
88	CDR3-77C	54.8	0.000	0.097	22.0	836.4	20909
89	CDR3-7C	54.8	0.000	0.097	19.3	451.9	11297
90	CDR3-9A	32.3	0.032	0.129	30.0	872.7	21819
91	CDR3-9B	51.6	0.000	0.032	21.3	803.3	20081
92	CDR3-9C	38.7	0.032	0.129	53.3	952.9	23822
93	CDR3-9D	45.2	0.000	0.000	27.3	705.2	17631
Mean		47.7	0.007	0.082	24.5	657.4	16435

Extraction of DNA from the final 93 clone set of 5000_RAD_ camel radiation hybrid panel was performed from the bulk cell pellet collected from two T150 flasks. The estimated yield of harvested cells varied from 6.1 million to 62 million among different hybrids, with a mean of 24.5 million/hybrid. The total DNA yield from these cells ranged from 164.2 µg to 1621.6 µg with a mean of 657.4 µg per hybrid (Table 1). The quality of extracted DNA from the final camel RH panel was high, with mean A260/280 and A260/230 ratios of 1.92 and 2.05 respectively. The total DNA yield from the camel RH panel was estimated to be sufficient for performing at least 4100 PCR reactions in duplicates.

To complement the standard 5000_RAD_ panel, we also generated a high irradiation dose (15000_RAD_) RH panel for mapping with increased resolution. A total of 238 well isolated hybrid clones were transferred into 24-well plates. 93 of 238 hybrids were further grown and screened with the same set of 44 markers in duplicate. Seven marker loci (CMS121, CMS15, GG_435, GG_1032, HTR3B, ATP6AP1 and AKAP12) that showed higher discordance were excluded and remaining 37 markers were considered for further analysis. Among the 93 high dose radiation hybrids, 20 (21.5%) had a mean RF less than 20%, 42 (45.2%) had a mean RF ranging from 20–50%, 16 (17.2%) had a mean RF ranging from 50–70% while 15 (16.1%) had a mean RF greater than 70%. The mean RF per hybrid ranged from 2.7% to 97.3% with an overall mean of 40.7%. Three hybrid clones with highest and lowest RF were dropped, leaving 90 clones in the panel with a mean RF of 39.9%. Further, screening of Y chromosome specific TR4520 and TR5720 markers showed a mean retention of 35.6% and 40.0% respectively. The mean retention of dromedary Y chromosome was in a similar range reported for bovine Y chromosome^29^.

Overall, the 5000_RAD_ camel radiation hybrid (RH) panel contains 93 hybrids and controls with an average retention frequency of 47.7%. This resource is immediately available for use to construct a camel RH map, which can assist in assembling camelid genomes to the chromosome level. The high-resolution 15000_RAD_ camel radiation hybrid (RH) panel contains 90 hybrids and controls with an average retention frequency of 39%, and is available to map complex regions such as the major histocompatibility complex (MHC) or the sex chromosomes.

## Discussion

With the introduction and subsequent evolution of next generation sequencing (NGS) technologies involving short and long reads, large volumes of genomic data can be produced in a short period of time and at a relatively low cost. However, *de novo* assembly of genomes to the chromosome level has remained a challenge due to highly fragmented short read assemblies and a lack of inexpensive scaffolding techniques. Radiation hybrid (RH) mapping has been proven to be a reliable approach for producing chromosome level maps for animals. High resolution whole genome RH maps played a pivotal role in obtaining chromosome level assemblies for the genomes of several mammalian and avian species that are currently listed in the ENSEMBL, UCSC and NCBI databases, e.g. human^30,31^, chicken^32^, cattle^33,34^, pig^14^, horse^35^ and goat^36,37^. Other mapping techniques such as interfacing NGS with optical mapping^38^, chromatin interaction based chromosome-scale (Hi-C) scaffolding^39^ and reference assisted chromosome assembly^40^ integrated with physical mapping^41^ are increasingly used to assemble genomes to chromosome level. However, recent reports indicate that radiation hybrid data is highly valuable when combining different sequencing and mapping procedures to produce highly accurate reference genome assemblies (e.g. goat^37^). Radiation hybrid data has been useful in resolving the conflicts related to misorientation of contigs within scaffolds, prediction of scaffold placements and orientation before final gap filling and polishing. Bickhart *et al*.^37^ reported that they were unable to find any other data set, apart from the RH map, that accurately predicted PGA (Hi-C based proximity guided assembly) scaffolds containing orientation errors to a high degree of accuracy.

Camelids are a group of species with strikingly similar chromosomal complement. The comparative chromosome painting and cytogenetic mapping data indicate that there are little or no inter-chromosomal rearrangements among camelid species^7,9^. However, as evidenced by comparative genomics of many closely related species, intra-chromosomal rearrangements like inversions and differences in heterochromatin distribution could be expected among the genomes of camelids. Nevertheless, the dromedary RH panel can form the basis for high resolution mapping and chromosome level assembly of all camelid species, once the reference assembly is created. The fibroblast cell line used for radiation hybrid fusion in the present study was purposefully established from an animal that already has a scaffolded whole-genome sequence assembly. Availability of the RH panel and whole-genome sequence from the same animal is expected to simplify the process of achieving a chromosome level assembly. This may be useful if low coverage survey sequencing is applied for characterizing the RH panel^42^, particularly in the absence of a camel specific SNP microarray for genome-wide typing. Further, metacentric and sub-metacentric chromosomes often represent challenge for the chromosome assembly as many mapping methods struggle with the positioning of centromeric regions. Even more, in case of dromedary, chromosomes 7,9,14,16,34 and X have euchromatin on short arms while the rest of the chromosomes have heterochromatin on the short arms^9^ (Supplementary Figure SF1). In these cases, RH map will be extremely useful for chromosome level assembly of the genome.

It has been previously shown that the use of a combination of RH panels constructed with different irradiation doses provides superior mapping results^14,15,31^. Here we used two contrasting doses of irradiation to make resourceful combination of RH panels: the standard for mammals at 5000_RAD_ and a high-resolution at 15000_RAD_. The 15000_RAD_ panel can be used to obtain a high-resolution map, to order markers in a genomic region of interest at the fine scale, and to resolve gene order in complex regions such as MHC. Furthermore, since the high resolution panel is derived from a male donor, it can be used for mapping the Y chromosome. However the 15000_RAD_ panel alone may not be sufficient to map the whole genome because of a likely fragmentation of linkage groups. On the other hand, a lower-resolution backbone map (5000_RAD_) is likely to produce whole-chromosome linkage groups suitable for chromosome assembly.

An important feature to note in camel-hamster hybrids was the high mean retention frequency (about one-third of hybrids had RF > 65%) observed per hybrid clone. The overall mean RF of the final set of 5000_RAD_ 93 hybrids (47.7%) was significantly higher than that of hybrids from most other domestic animal species (21–34.2%) produced under a similar radiation dose (5000_RAD_). The overall mean RF was 28% for the cattle RH panel (BovR5^43^), 21% for dog (RHDF5000^44^), 26% for horse (Equine RH5000^45^), 30.6% for pig (SSRH5000^46^), 25% for sheep (USUoRH5000^47^), 27.3% for river buffalo (BBURH5000^48^) and 34.2% for goat (CHRH5000^49^). Similarly, much lower overall mean RF of 21.9% and 23.6% was observed in the 6000_RAD_ chicken^50^ and duck^42^ panels respectively. Surprisingly, the dromedary camel panel followed the pattern of high retention observed in alpaca RH panel (Perelman *et al*. Unpublished). The reason for such high uptake and retention of camelid chromosomes by recipient cells is currently unknown.

Retention of donor genome in hybrids can be influenced by several factors, including but not limited to (i) fusion efficiency of donor and recipient cells, (ii) location of markers used for screening radiation hybrids, (iii) radiation dose, and (iv) integration and replication of donor chromosomes in recipient cells. The fusion efficiency (number of hybrid colonies per million irradiated donor cells fused) depends on the compatibility of structure and composition of membranes from donor and recipient cells^51^, compatibility of culture conditions (e.g. optimal temperatures for the growth of donor and recipient cells^42^), growth inhibiting effects of donor DNA integration/recombination with the recipient genome^52^, choice of selective marker (thymidine kinase or hypoxanthine phosphoribosyl transferase), ability of selective marker gene to be transcribed and translated in a rodent environment, and the ability of selective marker protein to function normally in hybrid cells so as to enable their survival^51^. In general, the shorter the evolutionary distance between donor and recipient species, the better the fusion efficiency. In mammals, the fusion efficiency ranged from 10 × 10^−6^ cells in dogs^44^, 22.4 × 10^−6^ cells in cattle^43^, 28.9 × 10^−6^ cells in goat^49^ and 37.3 × 10^−6^ cells in horse^45^, while the fusion efficiency in non-mammalian vertebrates was much lower ranging from 0.5–1 × 10^−6^ cells in zebrafish^53^ to 1.4 × 10^−6^ cells in chicken and 3.5 × 10^−6^ cells in duck^42^. In the present study, the fusion efficiency was estimated to be 588.9 × 10^−6^ cells for 5000_RAD_ panel, which is about 15–20 times higher than reported for other mammalian species. For the 15000_RAD_ panel, the fusion efficiency was 76.3 × 10^−6^ cells. Although the fusion efficiency dropped with higher dose of irradiation, it was still 2–3 times higher than reported for other mammalian species. Higher retention of camel genome observed in the hybrids might be partly explained by the higher fusion efficiency observed between irradiated camel fibroblasts and hamster cells.

The location of markers used for screening the hybrids can also play an important role in the estimation of the retention of donor genome. Markers in the centromeric regions of chromosomes often show higher retention frequencies as it has been reported in several species including the pig^54^, mouse^55^, rhesus macaque^56^, chicken and duck^42^. Probably, this could be due to the ability of centromere containing fragments to achieve replication in hybrid cells without being integrated into recipient chromosomes^56^. Although, the number of such “independent” non-integrated chromosomal fragments seems rather low based on few FISH experiments on hybrid clones^45,57,58^. In case of the chicken and duck, the retention frequency of markers located in micro chromosomes (that are often closer to centromeres) have been reported to be higher than those located in macro chromosomes^42,51^. However, in the present study, the markers were designed from sequences distributed throughout the dromedary camel genome (including genic regions) and most were not located near centromeres. Out of 44 genotyped markers, 28 had a known position on the chromosomes based on the alpaca genome map^9^ and on unpublished alpaca RH data: only 1 marker had a location close to a centromere and 6 markers were close to telomeres. We also hypothesize that the high retention frequency might be related to the dromedary genome composition, particularly the heterochromatin (some particular repeats) that make irradiated fragments of camel chromosomes “stick” together and efficiently get into the hamster cell. Both alpaca and dromedary have quite prominent heterochromatin blocks, unlike many other RH-mapped animals^59^ (Supplementary Figure SF1).

Interestingly, one third of the hybrids retained about 50% of camel genome, even at the high dose of irradiation. This clearly indicated the higher level of camel genome retention in radiation hybrids as compared to other mammalian species. The overall mean RF of the highly selected subset of hybrids derived from donor cells irradiated at a dose of 12000_RAD_ was reported to be 30.6% in cattle (88/204 hybrids^60^), 35% in pig (90/243 hybrids^61^) and 31.8% in sheep (90/208 hybrids^62^). Another possible explanation for higher donor retention could be the relatively better rate of integration between camel and hamster chromosomes in hybrids. Although donor DNA fragments can be maintained as additional independent chromosomes without being necessarily integrated into recipient genome, efficient integration can lead to higher retention rate^63^. Potentially, this could have occurred as a result of certain camel sequences that preferentially recombined with hamster chromosomes^56^.

Large quantities of DNA from radiation hybrid panels are generally required to type several thousand markers for building genome-wide maps using conventional PCR techniques^33,64–67^. This requires large-scale cultures leading to the spike in culturing cost, increased time and labor expenses. However, many other genotyping strategies like SNP microarrays^14,36^, survey sequencing based on NGS technologies, qPCR based Integrated Fluidic Circuits Dynamic Array^42^ have evolved recently that do not require large quantities of DNA, thus eliminating the need for clone expansion and intensive culturing. In the case of the dromedary radiation hybrids, high yield of DNA ranging from 164.2 µg to 1621.6 µg was produced within three passages of culture. Analysis of radiation hybrid panels derived from early passages not only results in higher retention frequencies, but also minimizes the occurrence of ambiguous genotyping results^52^. A highly stringent threshold was applied on discordance and difference rates for hybrid selection and hence the camel 5000_RAD_ RH panel is expected to have good stability with reduced variation in signal intensities during genotyping.

In conclusion, we herein report the construction of two radiation hybrid panels for dromedary. The camel 5000_RAD_ RH panel has a retention rate of 47.7%, which is close to the ideal frequency of 50% with good stability. A high yield of RH panel DNA can be used for more than four thousand PCR based genotyping reactions. The 15000_RAD_ camel panel has a mean retention frequency of 39.9% and is suitable for high-resolution mapping. These important genetic resources are available upon request for camel genome researchers and are expected to be highly helpful for constructing high resolution maps as well as building a camel genome assembly at the chromosome level.

### Availability of Data/Resource

The DNA for the two dromedary camel RH panels (5000_RAD_ and 15000_RAD_) is available upon email request to the corresponding author K.Periasamy@iaea.org or Official.mail@iaea.org.

## Electronic supplementary material

Supplementary Information

Supplementary Dataset1

## References

[1] FAOSTAT. http://faostat.fao.org/site/573/DesktopDefault.aspx?#ancor (2014).

[2] Hülsebusch, C. G. & Kaufmann, B. A. Camel breeds and breeding in northern Kenya, Kenya Agricultural Research Institute, Nairobi (2002).

[3] Watson EE, Kochore HH, Dabasso BH (2016). Camels and Climate Resilience: Adaptation in Northern Kenya. Human Ecology.

[4] Kebede, S., Animut, G. & Zemedu, L. The contribution of camel milk to pastoralist livelihoods in Ethiopia An economic assessment in Somali Regional State. IIED Country Report, IIED, London (http://pubs.iied.org/10122IIED) (2013).

[5] Elhadi YA, Nyariki DM, Wasonga OV (2015). Role of camel milk in pastoral livelihoods in Kenya: contribution to household diet and income. Pastoralism: Research, Policy and Practice.

[6] Wako, G. Economic value of camel milk in pastoralist communities in Ethiopia: Findings from Yabello district, Borana zone. IIED Country Report. IIED, London. http://pubs.iied.org/10119IIED (2015).

[7] Balmus G (2007). Cross-species chromosome painting among camel, cattle, pig and human: further insights into the putative Cetartiodactyla ancestral karyotype. Chromosome Research.

[8] Avila F (2014). Development and Application of Camelid Molecular Cytogenetic Tools. Journal of Heredity.

[9] Avila F (2014). A comprehensive whole-genome integrated cytogenetic map for the alpaca (*Lama pacos*). Cytogenetics and Genome Research.

[10] Avila F (2015). A cytogenetic and comparative map of camelid chromosome 36 and the minute in alpacas. Chromosome Research.

[11] The Bactrian Camels Genome Sequencing and Analysis Consortium. Genome sequences of wild and domestic Bactrian camels. *Nature communications* **3**, 1202 (2012). 10.1038/ncomms2192 PMC3514880 23149746

[12] Wu H (2014). Camelid genomes reveal evolution and adaptation to desert environments. Nature communications.

[13] Fitak RR, Mohandesan E, Corander J, Burger PA (2016). The *de novo* assembly and annotation of a female domestic dromedary of North African origin. Molecular Ecology Resources.

[14] Servin B, Faraut T, Iannuccelli N, Zelenika D, Milan D (2012). High-resolution autosomal radiation hybrid maps of the pig genome and their contribution to the genome sequence assembly. BMC Genomics.

[15] Bach LH (2012). A high-resolution 15,000_Rad_ radiation hybrid panel for the domestic cat. Cytogenetic and Genome Research.

[16] Holl, H. M. *et al*. *De novo* Assembly of a Dromedary Camel. In PAGXXV, San-Diego, CA, pp. W103, January 13–18 (2017).

[17] Stanyon R, Galleni L (1991). A rapid fibroblast culture technique for high resolution karyotypes. Italian Journal of Zoology.

[18] Wong PBY (2012). Tissue sampling methods and standards for vertebrate genomics. GigaScience.

[19] Westerveld A, Visser RPLS, Meera Khan P, Bootsma D (1971). Loss of human genetic markers in man-chinese hamster somatic cell hybrids. Nature New Biology.

[20] Page, J. E. & Murphy, W. J. Construction of Radiation Hybrid Panels. Phylogenomics, 422 of the series Methods in Molecular Biology, pp 51–64 (2008). 10.1007/978-1-59745-581-7_4 18629660

[21] Untergasser A (2012). Primer3 - new capabilities and interfaces. Nucleic Acids Research.

[22] Morgulis A (2008). Database indexing for production MegaBLAST searches. Bioinformatics.

[23] Chowdhary BP (2003). The first generation whole genome radiation hybrid map in the horse identifies conserved segments in human and mouse genomes. Genome Research..

[24] Amaral MEJ (2007). Construction of a river buffalo (*Bubalus bubalis*) whole-genome radiation hybrid panel and preliminary RH mapping of chromosomes 3 and 10. Animal Genetics.

[25] Barrett J (1992). Genetic mapping based on radiation hybrid data. Genomics.

[26] Lange K, Boehnke M (1992). Bayesian methods and optimal experimental design for gene mapping by radiation hybrids. Annals of Human Genetics.

[27] Lunetta KL, Boehnke M (1994). Multipoint radiation hybrid mapping: Comparison of methods, sample size requirements, and optimal study characteristics. Genomics.

[28] Jones HB (2008). Hybrid selection as a method of increasing mapping power for radiation hybrids. Genome Research.

[29] Liu W-S (2002). A radiation hybrid map for the bovine Y Chromosome. Mammalian Genome.

[30] Venter JC (2001). The sequence of the human genome. Science.

[31] Olivier M (2001). A high-resolution radiation hybrid map of the human genome draft sequence. Science.

[32] Leroux S (2005). Construction of a radiation hybrid map of chicken chromosome 2 and alignment to the chicken draft sequence. BMC Genomics.

[33] Everts-van der Wind A (2005). A high-resolution whole-genome cattle-human comparative map reveals details of mammalian chromosome evolution. Proceedings of National Academy of Sciences USA.

[34] Zimin AV (2009). A whole-genome assembly of the domestic cow, *Bos taurus*. Genome Biology.

[35] Wade CM (2009). Genome Sequence, Comparative Analysis, and Population Genetics of the Domestic Horse. Science.

[36] Du X (2014). An update of the goat genome assembly using dense radiation hybrid maps allows detailed analysis of evolutionary rearrangements in Bovidae. BMC Genomics.

[37] Bickhart DM (2017). Single-molecule sequencing and chromatin conformation capture enable *de novo* reference assembly of the domestic goat genome. Nature Genetics.

[38] Dong Y (2013). Sequencing and automated whole-genome optical mapping of the genome of a domestic goat (*Capra hircus*). Nat. Biotechnol.

[39] Burton JN (2013). Chromosome-scale scaffolding of *de novo* genome assemblies based on chromatin interactions. Nature Biotechnology.

[40] Kim J (2012). Reference-assisted chromosome assembly. Proceedings of National Academy of Sciences, USA.

[41] Damas J (2017). Upgrading short-read animal genome assemblies to chromosome level using comparative genomics and a universal probe set. Genome Research.

[42] Rao M (2012). A duck RH panel and its potential for assisting NGS genome assembly. BMC Genomics.

[43] Womack JE (1997). A whole-genome radiation hybrid panel for bovine gene mapping. Mammalian Genome.

[44] Vignaux F (1999). Construction and optimization of a dog whole-genome radiation hybrid panel. Mammalian Genome.

[45] Chowdhary BP (2002). Construction of a 5000Rad whole-genome radiation hybrid panel in the horse and generation of a comprehensive and comparative map for ECA11. Mammalian Genome.

[46] Hamasima N (2003). Construction of a new porcine whole-genome framework map using a radiation hybrid panel. Animal Genetics.

[47] Wu CH (2007). An ovine whole-genome radiation hybrid panel used to construct an RH map of ovine chromosome 9. Animal Genetics.

[48] Amaral ME (2008). A first generation whole genome RH map of the river buffalo with comparison to domestic cattle. BMC Genomics.

[49] Du XY (2012). A whole-genome radiation hybrid panel for goat. Small Ruminant Research.

[50] Morisson M (2002). ChickRH6: a chicken whole-genome radiation hybrid panel. Genetics Selection Evolution.

[51] Faraut T (2009). Contribution of radiation hybrids to genome mapping in domestic animals. Cytogenetics and Genome Research.

[52] Karere GM, Lyons LA, Froenicke L (2010). Enhancing radiation hybrid mapping through whole genome amplification. Hereditas.

[53] Kwok C (1998). Characterization of whole genome radiation hybrid mapping resources for non-mammalian vertebrates. Nucleic Acids Research.

[54] Liu W (2005). A 12,000-rad porcine radiation hybrid (IMNpRH2) panel refines the conserved synteny between SSC12 and HSA17. Genomics.

[55] Park CC (2008). Fine mapping of regulatory loci for mammalian gene expression using radiation hybrids. Nature Genetics.

[56] Karere GM, Froenicke L, Millon L, Womack JE, Lyons LA (2008). A high resolution radiation hybrid map of rhesus macaque chromosome 5 identifies rearrangements in the genome assembly. Genomics.

[57] Walter MA, Goodfellow PN (1993). Radiation hybrids: irradiation and fusion gene transfer. Trends in Genetics.

[58] Yerle M (1998). Construction of a whole-genome radiation hybrid panel for high-resolution gene mapping in pigs. Cytogenetics and Cell Genetics.

[59] Di Berardino D (2006). Cytogenetic characterization of alpaca (*Lama pacos*, fam. Camelidae) prometaphase chromosomes. Cytogenetic and Genome Research.

[60] Rexroad CE, Schlapfer JS, Yang Y, Harlizius B, Womack JE (1999). A radiation hybrid map of bovine chromosome one. Animal Genetics.

[61] Yerle M (2002). Generation and characterization of a 12,000-rad radiation hybrid panel for fine mapping in pig. Cytogenetics and Genome Research.

[62] Laurent P (2007). A 12,000-rad whole-genome radiation hybrid panel in sheep: application to the study of the ovine chromosome 18 region containing a QTL for scrapie susceptibility. Animal Genetics.

[63] McCarthy LC (1997). A First-Generation Whole Genome–Radiation Hybrid Map Spanning the Mouse Genome. Genome Research.

[64] Jann OC (2006). A second generation radiation hybrid map to aid the assembly of the bovine genome sequence. BMC Genomics.

[65] Davis BW (2009). A high-resolution cat radiation hybrid and integrated FISH mapping resource for phylogenomic studies across Felidae. Genomics.

[66] Hamasima N (2008). A new 4016-marker radiation hybrid map for porcine-human genome analysis. Mammalian Genome.

[67] Raudsepp T (2008). A 4,103 marker integrated physical and comparative map of the horse genome. Cytogenetics Genome Research.

