# Supplementary material for: Construction of two whole genome radiation hybrid panels for dromedary (Camelus dromedarius): 5000RAD and 15000RAD
Source: Sci Rep. 2018 Jan 31;8:1982. doi: 10.1038/s41598-018-20223-5 (PMC5792482; doi:10.1038/s41598-018-20223-5)
Supplement: Supplementary file 1 — Supplementary Information [file 41598_2018_20223_MOESM1_ESM.pdf]

Title : Construction of two whole genome radiation hybrid panels for dromedary (*Camelus dromedarius*): 5000RAD and 15000RAD

Authors : Polina Perelman [a,b], Rudolf Pichler [a], Anna Gaggl[a], Denis M. Larkin[c], Terje Raudsepp[d], Fahad Alshanbari[d], Heather M. Holl[e], Samantha A. Brooks[e], Pamela A. Burger[f] and Kathiravan Periasamy[a*]

Affiliations : [a]Animal Production and Health Laboratory, Joint FAO/IAEA Division, International Atomic Energy Agency, Vienna, Austria

[b]Institute of Molecular and Cellular Biology and Novosibirsk State University, Novosibirsk, Russia

[c]Department of Comparative Biomedical Sciences, Royal Veterinary College, University of London, London NW1 0TU, United Kingdom

[d]Texas A&M University, College Station, Texas, USA

[e]University of Florida, Gainesville, Florida, USA

[f]Research Institute of Wildlife Ecology, Vetmeduni Vienna, Austria

Corresponding author: Kathiravan Periasamy

Address : Animal Production and Health Laboratory, Joint FAO/IAEA Division of Nuclear Techniques in Food and Agriculture, International Atomic Energy Agency, Seibersdorf, Vienna, Austria

Email : K.Periasamy@iaea.org; kathirvet@yahoo.co.in

Telephone : 00431260028358

Supplementary Figure SF1. C-banding pattern of the CDR2 cell line used to construct the 5000_{RAD} panel. Note prominent centromeric blocks of heterochromatin.

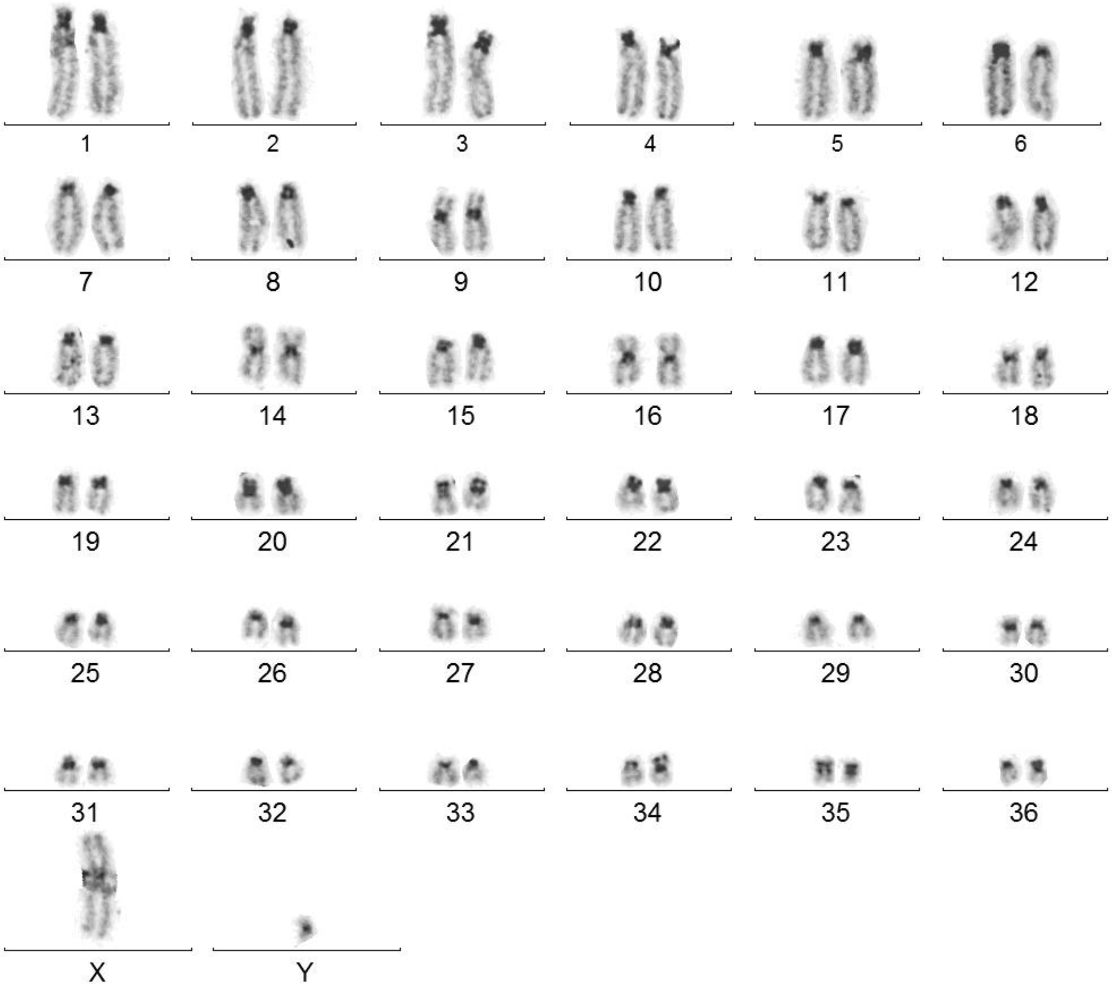

Supplementary Table ST1. Oligo sequences, template genome details and reaction conditions for custom-designed markers used to screen camel-hamster radiation hybrids

S. No	Marker Name*	Accession No.	Scaffold	Start	End	Primer-Forward	Primer-Reverse	Annl Temp (°C)	Amplicon size	Alpaca chromosome*
1	GG_797	JWIN01033025	8668383	244476	244672	AGTCTGGAAAAGCAGCCTCA	TGAGGCCAACTCAGGATAGG	57	146	LPA1
2	KCNIP4	JWIN01034885	8670243	236867	237034	GACACTTCATAAGAAGGGACATTACA	AGCCCATTAGGAAGGTAACTCA	57	114	LPA2
3	AFM	JWIN01034683	8670041	703112	703326	TCTGGCATTTCTCTTCAGGA	GGGCTTCCACAAAAGCATAA	57	142	LPA2
4	VOLP10	JWIN01030202	8665560	2127106	2127471	TTCCAGTCCTTTCTCCTTTCC	TCATCTGAAACAAGGCTTCCT	57	216	LPA2
5	GG_905	JWIN01031128	8666486	18886	19077	GCCAAATGTGGCTATTTGTT	TTCTTCCTCAAGAGTTTCTGTGG	57	127	LPA3
6	GNAQ	JWIN01029304	8664662	2134045	2134228	CCTAATTCCCAGCAGTGACG	TCAAGTCAAGCCTGATGGTG	57	112	LPA4
7	CSNK1G1	JWIN01033098	8668456	531134	531361	TGGCCAGCACTGAATAATGA	AGCGCATATGCCACTCTCTT	57	149	LPA6
8	CMS121	JWIN01030491	8665849	1206525	1206888	TCTTGCTCAGTCATCTGTAAATCC	GGCAAGAGAACTGGTGAGGA	57	185	LPA7
9	GG_1484	JWIN01032354	8667712	1609769	1609957	ATGGCGATGAAAATCCACTC	TTTGAGGAAGTGCCATGATTC	57	110	LPA7
10	AKAP12	JWIN01033438	8668796	453208	453840	CTGGAACTGGAGTTGGCAAT	AAGGCTTCCCAGGACTTCAC	57	211	LPA8
11	CMS13	JWIN01033684	8669042	109907	110202	GGTCTCCTTGCTTGGCTCTT	TGGCATCCACTGATTTTTGA	57	190	LPA8

12	CYP2S1	JWIN01034991	8670349	947794	948266	CCTTCCCTTCCCCTACAGTC	CCACCATCCCACGAAACTAC	57	200	LPA9
13	CMS15	JWIN01033713	8669071	1437790	1437956	CCAGAAAGGCAGTTTCTCCA	GGTTCCCAGATGTAATTATGGATT	57	123	LPA10
14	LDB3	JWIN01034929	8670287	304646	304925	AAAAGAATTCACACAGGCAAAA	TGACTTGTGTCTGCAGTGTTTG	57	183	LPA11
15	PRKG1	JWIN01031962	8667320	1271704	1271903	CAATGGAAACCAGAGGGATG	GGATTACCCACCCCTGAAAT	57	184	LPA11
16	KITL	JWIN01031144	8666502	195976	196146	GTGTTCTTCCATGCACTCCA	AGATGGTGGCACAGTTGTCA	57	143	LPA12
17	HEYL	JWIN01034693	8670051	89223	89419	TGGGGTAAGCAAGAGAGGAG	CCTTCTTTCCCACCTCAACA	57	197	LPA13
18	CMS9	JWIN01035050	8670408	2109894	2110491	GCCTGGTTCTCTCAACCAAA	GGCAAGTTTCCTAAATTGCTCT	57	202	LPA14
19	GG_435	JWIN01029495	8664853	358376	358531	AAATGAATCCATTCCGCAAA	TGTCACACAAAATGACTCCAGA	57	112	LPA14
20	PAIP2B	JWIN01035346	8670704	60230	60427	GAGGACAGATGTGCAGTGTGA	AAGTGAGATGGAGGGGGAGT	57	156	LPA15
21	JMJD6	JWIN01031135	8666493	2860600	2860741	GCTCACCCCTGAGCAGATAA	AGCTCGGACCAGAGAGGATT	57	139	LPA16
22	MITF	JWIN01030173	8665531	272307	272506	GTGTTTCCTCTGTGCCAGGT	CAGTGAGGCATACACCTTCC	57	160	LPA17
23	VAPB	JWIN01032345	8667703	5017661	5018229	TCATAGGGAAGATCGCCTTG	TACCTGTGTGTTGGGGTGTG	57	192	LPA19
24	GNL1	JWIN01034489	8669847	68658	69236	TATTTGCCAGTTGCTGCTCA	TCCAGACCTGTCCTCCACTC	57	202	LPA20

25	KLHDC9	JWIN01035381	8670739	1722576	1722773	ATTGCCAAGCCTCTGTTTTG	TTACCGTTTCCCTTGCAAAT	57	168	LPA21
26	GG_1032	JWIN01029458	8664816	982497	982695	ACGGGCTTTTTGTTGATCC	CGGTCCCACAACTCAAAAA	57	179	LPA22
27	KISS1	JWIN01034763	8670121	334555	334753	CGCCCCTACTCTGGGTATAA	TCCACTCATCTTTTGCCACA	57	175	LPA23
28	GG_984	JWIN01035356	8670714	563275	563474	GCGTATGAGAATGGTTCGATG	AGGACCTGCTTGTGAACTGC	57	156	LPA24
29	GG_1030	JWIN01031455	8666813	248212	248410	CAAGGCTAGGTGGTGATGCT	TGACAGCATATTTAGGAATGAGGA	55	149	LPA26
30	CMS25	JWIN01030760	8666118	604909	605148	TTCCTCTGGGATCTGTGCTT	GATGCACTGCTCAGATGGTC	57	201	LPA27
31	IL1A	JWIN01034013	8669371	1521463	1521688	TTGGCAATGGCTTCTAGGTC	AGCCACTTCAAGAGGACTGC	57	184	LPA28
32	RALYL	JWIN01031426	8666784	491256	491382	GATGAAGAAGACCCGCTGAC	CTCCCCGCGCAGTAATTC	57	121	LPA29
33	lgnuc681	JWIN01030866	8666224	728377	728749	CACATTTCCATCCCCTGACT	AAAAGCTCTGCCTTGTTCCA	55	191	LPA30
34	GATA4	JWIN01033843	8669201	201984	202176	GCTCTGAAAGCAACCAGCTT	AGCACTCTCAGGGTTGACCA	55	156	LPA31
35	GNB1L	JWIN01032640	8667998	499846	500091	GACACTGATGCGCTGATCC	AACTCACCAGCTCACCAACC	57	220	LPA32
36	HTR3B	JWIN01033805	8669163	1100776	1100966	CACCTGCTACTGGCAGTCATT	TTGCAGACATGGAAAATCACA	55	134	LPA33
37	VOLP32	JWIN01032672	8668030	1446691	1447232	CTCCCTGATCCTGCATCTCT	ACAGAAGCCACCAGGAGGT	57	264	LPA34
38	GG_498	JWIN01033073	8668431	940616	940817	GCGGGTCTTTGGAAGAAAAG	CCAGGAAACCTTTCCACTGT	57	125	LPA34

39	CREM	JWIN01034202	8669560	142554	142864	GAAGCTTGTGACCCTCCAGA	CTTCCGACGTCGACACTCTT	57	200	LPA35
40	Sc395	JWIN01034444	8669802	177861	178570	GGCCTTTCCTGATCACTCCC	CCAAGACCCTCAGAGCTGTT	57	136	LPA36
41	ATP6AP1	JWIN01031654	8667012	40424	40752	GCTCCCTCCTCAGGCTTTAT	TGGAGGTCCTTTCCTTCTCA	57	196	LPAX
42	GG_1083	JWIN01033597	8668955	609478	609689	ATGCCAAGCTATGCCTTGAT	TGACTCAGGCTAGCTCACTGAT	55	214	LPAX
43	ZXDA	JWIN01030927	8666285	4257	4460	TTGGCATAAATTTGCCGTTT	GTTCAACAGAGTCCCCTGGA	55	161	LPAX
44	VOLP67	JWIN01035330	8670688	28840	29322	TGATCCAGGTGGAGGGTCTA	AGCGGAGTCAAGAAGGTGAA	55	156	LPAX
45	TR4520	NW011591851	899	156649	157372	GCCTTGCTGGAGCTCTTAAA	ACAGTACCAGGCCTCCTCCT	58	724	LPAY
46	TR5720	NW011591691	763	17491	18806	TTAGAGCTGAGCGCTGTGAA	CCAAGACGAAGATGTGCTGA	58	1316	LPAY

* Location of markers in Alpaca genome largely matches with their respective location in dromedary genome

Supplementary Table ST2. Details of final scoring based on electrophoretic results from two PCR screenings of camel-hamster radiation hybrids

PCR1	PCR2	Combination category	Final Score
1	1	Identical	Positive
1	2	Different	Positive
2	1	Different	Positive
3	1	Discordant	Discordant
1	3	Discordant	Discordant
2	2	Identical	Positive
2	3	Different	Negative
3	2	Different	Negative
1	0	Different/Discordant	Discordant
0	1	Different/Discordant	Discordant
2	0	Different	Negative
0	2	Different	Negative
3	3	Identical	Negative
3	0	Different	Negative
0	3	Different	Negative
0	0	Identical	Negative
